# EPIDEMIOLOGICAL PROFILE OF PATIENTS WITH TIBIA DIAPHYSIS FRACTURE TREATED AT A TERTIARY LEVEL HOSPITAL

**DOI:** 10.1590/1413-785220243205e279748

**Published:** 2024-10-28

**Authors:** José Carlos Baldocchi Pontin, Ana Paula Cortes Damasceno, Helder Joel Moreira de Souza, Isadora Salvador Rocco, Orlando Copetti Fração, Fabio Teruo Matsunaga

**Affiliations:** 1. Universidade Federal de São Paulo, Escola Paulista de Medicina, Departamento de Ortopedia e Traumatologia, Sao Paulo, SP, Brazil.; 2. Universidade Federal de São Paulo, Escola Paulista de Medicina, Programa de pós-graduação da Disciplina de Cardiologia, Sao Paulo, SP, Brazil.

**Keywords:** Fracture, Tibia, Orthopedics, Fratura, Tíbia, Ortopedia

## Abstract

Objective: To outline the epidemiological profile of tibial fractures treated in a tertiary hospital and explore associations between the characteristics of the fractures and the clinical outcome of postoperative complications. Methods: Retrospective cross-sectional study involving adult patients diagnosed with tibial fractures who underwent surgical and/or conservative treatment in a tertiary hospital between January 2019 and December 2021. The variables sex, age, mechanism of injury, type and classification of fracture, associated injuries, personal history, length of hospital stay, surgical treatment, post-surgical complications (infections, loss of synthesis material, surgical wound dehiscence) and death. Results: The sample consisted of 100 individuals, with an average age of 35.8 years, 86% of whom were male, with a higher prevalence of motorcycle accidents. The most common treatment was intramedullary stem, and type C fractures, which are more complex, were more associated with complications. Conclusion: Given the predominance of motorcycle accidents involving young people, there is a need for intervention in accident prevention policies, aiming to reduce the incidence, as well as the morbidity and mortality, of these individuals and, consequently, to reduce costs to the health system. ***Level of evidence III, Retrospective Study.***

## Resumo

Objetivo: Traçar o perfil epidemiológico das fraturas de tíbia tratadas em um Hospital de nível terciário e explorar associações entre a caraterística da fratura e o desfecho clínico de complicações pós-operatórias. Métodos: Estudo transversal retrospectivo, envolvendo pacientes adultos, com diagnóstico de fratura de tíbia que realizaram tratamento cirúrgico e/ou conservador em um hospital de nível terciário entre janeiro de 2019 e dezembro de 2021. Foram analisadas as variáveis sexo, idade, mecanismo de lesão, tipo e classificação de fratura, lesões associadas, antecedentes pessoais, tempo de internação hospitalar, tratamento cirúrgico, complicações pós cirúrgicas (infecções, perda do material de síntese, deiscência de ferida operatória) e óbito. Resultados: A amostra foi composta por 100 indivíduos, com média de idade de 35,8 anos, sendo 86% do gênero masculino, com maior prevalência de acidente motociclístico. O tratamento mais realizado foi a Haste intra-medular e as fraturas do tipo C, mais complexas, foram mais associadas às complicações. Conclusão: Diante da predominância de acidentes motociclísticos envolvendo o público jovem há necessidade de intervenção de políticas de prevenção de acidentes, visando diminuir a incidência, bem como a morbidade e mortalidade desses indivíduos e consequentemente a diminuição de custos ao sistema de saúde. ***Nível de evidência III, Estudo Retrospectivo.***

## INTRODUCTION

 Traumatology of the musculoskeletal system is an important problem in public health as it accounts for a large part of hospital care and is responsible for high morbidity and mortality rates. ^1^ ^,^ ^2^

 Diaphyseal tibial fractures are the most common among fractures of the long bones. In North America, around 300,000 fractures occur per year, and in Brazil around 50,000 per year. ^1^ Tibial fractures usually occur in two forms: spiral fractures, which are more frequent in people aged over 50 years and result from low-energy trauma, and transverse and/or comminuted fractures, which are more related to high-energy trauma in people aged over 30 years. Low-energy trauma is more related to falls from one’s height and sports injuries, while high-energy trauma is more associated with automobile accidents. ^2^

 The most used classification is the Orthopaedic Trauma Association/Arbeitsgemeinschaft für Osteosynthesefragen (OTA/AO), which considers the mechanism, location and energy of the trauma. Type “A” stands for simple fractures, type “B” for fragmented fractures with wedges and type “C” for complex multifragmented fractures. ^3^ ^,^ ^4^ Due to force transmission mechanisms and local anatomical characteristics, diaphyseal tibial fractures can have some complications and association with other injuries, such as compartment syndrome, ankle injuries, extension of injury to the tibial plateau, knee ligament injury, among others, which can trigger greater functional impairments and risks. ^1^ ^,^ ^2^

 The most used diagnostic method is radiography. In cases in which there is a suspicion of associated joint and/or ligament injuries, computed tomography or even, in some cases, magnetic resonance imaging is recommended. ^5^ ^,^ ^6^

 Treatment for diaphyseal tibial fractures can be conservative or surgical, with the latter being increasingly used in orthopedic practice. There are different options of surgical treatment, ^1^ ^,^ ^3^ ^,^ ^4^ which can take into account factors such as fracture type, age, among others, aiming at fracture stability, rehabilitation and an early return to activities, in addition to avoiding postoperative complications. ^4^ ^-^ ^6^

 Since traumatology of the musculoskeletal system comprehends an important part of hospital care and accounts for high rates of morbidity and mortality, especially related to postoperative complications, ^4^ ^,^ ^6^ it is very important to properly understand the current epidemiological situation of this condition in our environment. 

In Brazil, few epidemiological studies on this topic have been published in recent years, and there is currently no basis for a better understanding of the impact of tibial fractures on the health system and, consequently, for the development of preventive strategies to reduce the risk of comorbidities and complications. Thus, the objectives of this study were to outline the epidemiological profile of ankle fractures treated in a tertiary hospital and to explore associations between fracture characteristics and clinical outcomes of postoperative complications.

## MATERIAL AND METHODS

This is a retrospective study conducted by searching the electronic Patient Records System (PRS) for hospital admission records of patients with diaphyseal tibial fractures who were hospitalized and treated between January 2019 and December 2021. Records with incomplete data, patients under 18 years of age or transferred to other hospital services were excluded.

All the information used in this project was collected after the participants signed the Informed Consent Form (ICF) issued by the research team. This study was registered and approved by the Research Ethics Committee, with registration in Plataforma Brasil under CAEE number: 68883423.9.0000.5505, opinion 6.297.715.

The variables sex, age, mechanism of injury, fracture type, fracture classification, associated injuries and personal history were recorded. The analyzed outcomes were divided into primary, postoperative complications (infections, loss of synthesis material, wound dehiscence) and death; and secondary, length of hospital stay and surgical treatment variables.

### Statistical analysis

Categorical data were expressed as relative frequency (%), continuous data were expressed as mean and standard deviation, and discrete data were expressed as median and quartiles 25-75%. Firstly, a descriptive analysis was conducted to verify the prevalence and distribution of clinical, anthropometric and surgical characteristics of patients.

 To explore the association between fracture types “A”, “B” or “C” and the clinical outcomes, a generalized linear model was performed with the distributions linear, *gamma* and logistics, according to the nature of the dependent variables. The postoperative complication outcome was determined by the existence of at least one clinical event considered not expected in the postoperative period, such as loosening of surgical material, need for reoperation, surgical site infection, pneumonia, associated fracture and cardiorespiratory arrest. The adopted level of statistical significance was p < 0.05. The software used was JAMOVI ( *Version* 1.6.23.0). 

## RESULTS

 The data related to sociodemographic characteristics are shown in Table 1 . The study involved 100 patients: 86% of the participants were male and 14% female, with a mean age of 35.8 ± 14.6 years. 

**Table 1.** Sample distribution by gender and age.

Gender	n	%	Mean	M	Ma
Female	14	14.00	45.00	18	72
Male	86	86.00	33.93	18	71
Total	100	100.00	35.82	18	72

Legend: n - number of patients; % - percentage; M - minimum; Ma - maximum

Table 2 presents the data regarding the AO classification of fractures, with the most frequent fractures being the simple type (A), followed by wedge (B) and multifragmented fractures (C). A higher prevalence of simple type (A) fractures was found, with 58% of all fractures. 

**Table 2.** Distribution of diaphyseal tibial fractures as classified by the *Orthopaedic Trauma Association / Arbeitsgemeinschaft für Osteosynthesefragen* .

Fracture type	N	Percentage (%)
A1	22	22.00
A2	16	16.00
A3	20	20.00
B1	2	2.00
B2	16	16.00
B3	4	4.00
C1	1	1.00
C2	10	10.00
C3	9	9.00

Legend: N - number of patients, A1 (spiral), A2 (oblique), A3 (transverse), B1 (spiral wedge), B2 (flexion wedge), B3 (fragmented wedge), C1 (comminuted spiral), C2 (Segmental) and C3 (crushing)

 Among the mechanisms of injury, automobile traumas stood out, followed by falls (from one’s height or flat floor), pedestrian collisions and sports practice, respectively ( Table 3 ). Of the total, 6.00% could not report their mechanism of trauma. 

**Table 3.** Mechanism of injury.

Causes	N	Percentage (%)
Automobile accident	62	62.00
Pedestrian collision	14	14.00
Fall	15	15.00
Sports practice	3	3.00
Could not report	6	6.00

Legend: N - number of patients

 The most used treatment was the intramedullary stem (IM), followed by locking plates, conservative treatment and treatment with Ilizarov-type linear or ring external fixators ( Table 4 ). 

**Table 4.** Treatment

Treatment	N	Percentage
External fixator	4	4.00
Intramedullary stem	83	83.00
Plates and screws	8	8.00
Conservative treatment	5	5.00

Legend: N - number of patients

 Considering the classification of fractures into types A, B and C, it was found that fracture severity was a determining factor in the outcome of postoperative complications. Patients with a type C fracture were 4.9 times more likely to develop postoperative complications compared to those with a type A fracture (OR = 4.88 [95% CI = 1.61 – 15.46], p = 0.006, Table 5 ). No difference was found when comparing patients who evolved with a type B fracture compared to types A and C. 

**Table 5.** Odds ratio of clinical outcomes by group.

Postoperative complications						
Comparison of groups			OR(B)	CI 95%	Z	P value
C	-	A	4.88	1.61 – 15.46	2.77	0.006*
B	-	A	0.73	0.15 – 2.70	-0.45	0,655

Notes: OR - Odds ratio, CI - Confidence interval

**Figure 1.**
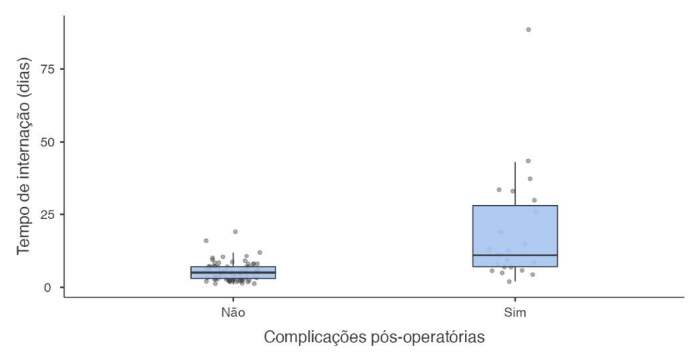
Relationship between length of stay and complications

 The mean length of hospital stay was 6 (3 - 8.75) days for patients with a type A fracture, 5 (3-7) days for patients with a type B fracture, and 6 (4 - 10.3) days for patients with a type C fracture. There was no association between fracture type and length of hospital stay (Kruskal Wallis = 1.42, df = 2, p = 0.491). Individuals who developed complications in the postoperative period had a longer hospital stay compared to those who did not have complications (Mann-Whitney U = 288, p < 0.001, Figure 1 ) 

No deaths occurred during this study.

## DISCUSSION

 One hundred patients with diaphyseal tibial fractures were studied, predominantly young adult males. This predominance of males has also been reported in other studies, such as Grecco’s ^7^ , in which 73.7% of the sample was composed of men. The mean age of the patients in this study was 35.82 years (18-72 years), which corroborates other studies found in the literature, ^8^ ^,^ ^9^ such as those by Ali ^8^ and Grecco ^7^ , who found mean ages of 38 and 32 years respectively. 

 Regarding fracture classification, simple (type A) fractures were the most found type, like the data from other studies that report a higher prevalence of simple fractures, also in hospitalized individuals. ^8^ ^,^ ^9^

 The higher incidence in working-age men is directly linked to those previously mentioned factors, especially as regards the mechanism of injury, usually of high energy. ^10^

 The literature notes that there are several methods of treatment for diaphyseal tibial fractures, as well as discussions favoring one method or another. A study conducted by Canadian surgeons showed that 80% of them treated diaphyseal tibial fractures surgically. ^6^ Nevertheless, in a conference of the Orthopaedic Trauma Association, 70% of orthopedic surgeons opted for surgical treatment of this injury. Over the years, conservative treatment has become less common due to the risks of non-consolidation and possible displacement of the fracture during treatment. ^5^ ^,^ ^6^

 The intramedullary stem is considered by many surgeons to be the most effective treatment for these fractures. ^6^ ^,^ ^10^ At this point, depending on when the study was conducted, the literature was conflicting. In the study by Grecco et al., ^7^ of the 179 diaphyseal tibial fractures, 86 underwent treatment with cast immobilization, 71 with external fixators and cast immobilization, 17 with plates and screws, and 5 with external fixators only. On the other hand, the study by Vieira Jr. et al. ^10^ showed a divergent situation, in which of the 123 fractures studied, 37.5% were definitively treated with dynamic compression plates (DCP), 20.3% with bridge plating and intramedullary stems, 15.6% with linear external fixators and 6.3% with Ilizarov-type ring external fixators. ^10^

 The mean length of hospital stay in this study was 8.2 days, a considerably lower number compared to data from other reported authors, who obtained a mean length of hospital stay of 26.61 days. ^11.^ The literature indicates that length of hospital stay is directly related to the number of procedures to which a patient is submitted. ^12^

 As in other studies, simple fractures (42A) were the most found type, while the wedge fractures (42B) and the complex, multifragmented fractures (42C) shown were not similar, but had no statistical significance. ^12^

 Vieira Junior et al., in a retrospective, descriptive and analytical study of a tertiary hospital, showed that the mean hospital stay for patients with a type A fracture was 25.5 days, with 16.8 days for type B and 48.3 days for type C. However, there was no statistical significance. ^10^

 Because it has a small range of muscle protection in its anterior part, the tibia is commonly fractured in an exposed way; and when evaluated between the mechanisms (high and low energy), this percentage varies between 12 and 47%, but when evaluated only in high energy traumas, the percentage increases to approximately 63%. ^2^ ^,^ ^5^ ^,^ ^13^ Similarly, when evaluating the number of hospital days in the literature, considering patients who had or did not have associated complications in the fracture, the length of hospital stay changes, resulting in a mean of 45.57 days for patients with associated injuries and 16.68 days for patients without associated injuries. ^10^ ^,^ ^11^

No topics were found in the literature regarding the death of patients after complications related to diaphyseal tibial fractures, nor to physiotherapy services being offered to, and engaged by, patients with diaphyseal tibial fractures after hospital discharge.

 The Ministry of Health warns that automobile accidents are the second largest cause of external deaths in Brazil. In 2017, 35.3 thousand people lost their lives due to a kind of automobile accident. It also reports that approximately BRL 260 million are spent annually on hospitalization of injured persons alone. In addition, those accident victims also cease to contribute to the state, meaning that they will not pay back what was spent on them, which strongly contributes to overburdening public services. ^14^

This study reinforces relevant data on diaphyseal tibial fractures with a robust number of participants. It corroborates important aspects regarding the proper care of these injuries, which will allow us to continuously improve treatment and obtain increasingly favorable outcomes.

## CONCLUSION

Diaphyseal tibial fractures had a higher incidence in males aged between 20 and 39 years. The most frequent cause was automobile accidents, mostly causing a simple (type A – AO) fracture, while patients with a type C fracture were more likely (4.9 x) to develop complications. Surgical treatment using an intramedullary stem was the most common method, accounting for 83% of cases, with a mean length of hospital stay of 8.42 days.
